# Supplementary material for: Sea Buckthorn Flavonoid Extracted by High Hydrostatic Pressure Inhibited IgE-Stimulated Mast Cell Activation through the Mitogen-Activated Protein Kinase Signaling Pathway
Source: Foods. 2024 Feb 12;13(4):560. doi: 10.3390/foods13040560 (PMC10887968; doi:10.3390/foods13040560)
Supplement: Supplementary file 1 [file foods-13-00560-s001.zip › foods-2837977-supplementary.pdf]

Figure S1

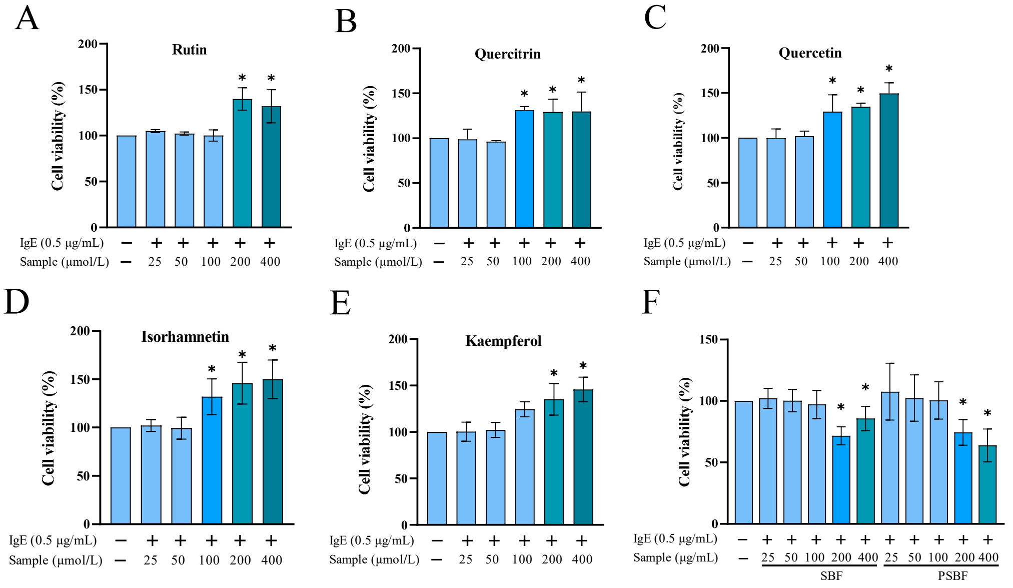

Figure S1. Five compounds (A-E) and cytotoxic effect of SBF, PSBF (F) on RBL-2H3 cells. Results are expressed as the mean ± SD of three independent experiments. (*p<0.05).

Table S1 The elution procedure of the mobile phase

Elution time (min)	Solvent A: acetonitrile (%)	Solvent B: 0.4% phosphoric acid aqueous solution (%)
0	10	90
7	15	85
10	17	83
25	17	83
27	18	82
31	19	81
34	19	81
38	26	74
42	26	74
55	50	50
60	10	90

Table S2 Box-Behnken design and response values for the flavonoid amount of sea buckthorn

Runs	A Pressure (MPa)	B Pressure-holding time (min)	C Ethanol concentration (%)	D Liquid-to-solid ratio (mL/g)	Y Flavonoid amount (mg/g)
1	1	0	1	0	3.58
2	1	0	0	1	3.20
3	1	1	0	0	4.00
4	1	0	0	-1	3.51
5	1	-1	0	0	3.58
6	1	0	-1	0	3.05
7	-1	-1	0	0	3.20
8	-1	0	0	1	3.19
9	-1	1	0	0	3.42
10	-1	0	-1	0	3.01
11	-1	0	1	0	2.81
12	-1	0	0	-1	2.81
13	0	-1	0	-1	4.21
14	0	-1	0	1	4.40
15	0	0	-1	-1	3.51
16	0	1	0	1	4.21
17	0	0	-1	1	3.80
18	0	-1	1	0	4.40
19	0	1	1	0	4.40
20	0	0	1	1	3.81

21	0	1	0	-1	4.39
22	0	1	-1	0	4.02
23	0	-1	-1	0	3.72
24	0	0	1	-1	4.01
25	0	0	0	0	5.09
26	0	0	0	0	4.92
27	0	0	0	0	4.88
28	0	0	0	0	5.07
29	0	0	0	0	4.97
